# Psychometric properties of Spanish-language adult dental fear measures

**DOI:** 10.1186/1472-6831-8-15

**Published:** 2008-05-12

**Authors:** Trilby Coolidge, Mark A Chambers, Laura J Garcia, Lisa J Heaton, Susan E Coldwell

**Affiliations:** 1 Dental Public Health Sciences, University of Washington, Seattle, WA, USA; 2 School of Dentistry, University of Washington, Seattle, WA, USA; 3 Heritage University, Toppenish, WA, USA

## Abstract

**Background:**

It would be useful to have psychometrically-sound measures of dental fear for Hispanics, who comprise the largest ethnic minority in the United States. We report on the psychometric properties of Spanish-language versions of two common adult measures of dental fear (Modified Dental Anxiety Scale, MDAS; Dental Fear Survey, DFS), as well as a measure of fear of dental injections (Needle Survey, NS).

**Methods:**

Spanish versions of the measures were administered to 213 adults attending Hispanic cultural festivals, 31 students (who took the questionnaire twice, for test-retest reliability), and 100 patients at a dental clinic. We also administered the questionnaire to 136 English-speaking adults at the Hispanic festivals and 58 English-speaking students at the same college where we recruited the Spanish-speaking students, to compare the performance of the English and Spanish measures in the same populations.

**Results:**

The internal reliabilities of the Spanish MDAS ranged from 0.80 to 0.85. Values for the DFS ranged from 0.92 to 0.96, and values for the NS ranged from 0.92 to 0.94. The test-retest reliabilities (intra-class correlations) for the three measures were 0.69, 0.86, and 0.94 for the MDAS, DFS, and NS, respectively. The three measures showed moderate correlations with one another in all three samples, providing evidence for construct validity. Patients with higher scores on the measures were rated as being more anxious during dental procedures. Similar internal reliabilities and correlations were found in the English-version analyses. The test-retest values were also similar in the English students for the DFS and NS; however, the English test-retest value for the MDAS was better than that found in the Spanish students.

**Conclusion:**

We found evidence for the internal reliability, construct validity, and criterion validity for the Spanish versions of the three measures, and evidence for the test-retest reliability of the Spanish versions of the DFS and NS.

## Background

According to the U.S. Bureau of Census statistics estimate for July 1, 2005, Hispanics comprise the largest ethnic minority in the United States [1]. More than 15% of the United States population is Hispanic, and more than 12% of the United States population aged 5 and older speaks Spanish at home [2,3]. Most Hispanics in the United States are of Mexican origin [4]. Oral health disparities are prevalent among this growing population [5]. For example, the 2000 Surgeon General's report on oral health cited statistics indicating that Hispanic adults were between two and three times more likely to have untreated caries compared with non-Hispanic whites [6]. Notably, these differences remain when employment status and/or income are controlled, indicating that financial disparities may not explain the higher caries rates. Since dental fear is known to contribute to dental avoidance [7], and since it appears to be found in every culture in which it has been studied [8], it is likely that dental fear may also be found in Hispanics in the United States.

To date, little is known about dental fear in Hispanics. One study found that dental fear was not a significant factor in explaining why Hispanics in the Southwest did not utilize dental services [9]. Another study, which assessed Mexican-American migrant farmworkers presenting for health care at a Midwestern clinic, found that 5% stated that fear of dental work had kept them from being able to go to a dentist [10]. However, the study did not use a standard measure of dental fear, and the design makes it difficult to generalize the findings. A third study of Californians found that 7.7% of Hispanics who had not been to a dentist in the past year stated that fear had been the primary reason for their decision [11].

The report of a 2004 conference which focused on Hispanic oral health highlighted the need for the development of reliable and valid measures for the Hispanic population [5]. Measures of dental fear appropriate for the Hispanic population would permit better epidemiological research, as well as help in the evaluation of dental fear treatments.

The two most frequently used measures of overall dental fear in adults are Corah's Dental Anxiety Scale (DAS) [12] and Kleinknecht and colleagues' Dental Fear Survey (DFS) [13]. Both were originally developed in English. The original DAS is a 4-item questionnaire, asking respondents to rate their anxiety as they imagine approaching four dental stimuli, such as contemplating going to the dentist tomorrow. Each item is answered on a 5-point scale, so that scores may range from 4 (no fear) to 20 (highest level of fear). A Spanish language version was developed for Puerto Ricans [14]; this version has also been used in Costa Rica [15] and Spain [16]. One group has also reported on a Spanish translation used in Spain [17,18]. Finally, one group used a Spanish translation in a study based in California; however, no psychometric information was provided [19].

The Modified Dental Anxiety Scale (MDAS) [20] was developed to improve the psychometrics and content validity of the original DAS. It consists of five items, and total scores may range from 5 (no fear) to 25 (highest level of fear). The MDAS has been found to be reliable and valid in several samples from England, Scotland, Wales, Ireland, Finland, Dubai, Brazil, and Turkey [20-24]. While the DAS is considered to be reliable and valid [25], the MDAS is considered to be an improvement over the original DAS [26]. To our knowledge, no Spanish version of the MDAS has been developed. Therefore, we elected to perform our own translation of this measure.

The original DFS was a 27-item questionnaire [27], which was subsequently reduced to the current 20-item version [25]. The items assess anxiety associated with a number of stimuli such as sitting in the dental chair and seeing the drill. Other items assess physiological changes, such as muscle tension and rapid heart beat. Two items assess avoidance due to fear, and one item asks for an overall rating of fear. Each item is rated on a 5-point scale. Possible scores range from 20 (no fear) to 100 (highest level of fear). The DFS has been found to be reliable and valid in samples of college students, general dental patients, and fearful dental patients [25], and has been translated into a number of languages, including Danish, Swedish, Norwegian, Hungarian, Brazilian, Turkish, Chinese and Malay [28-34].

One group has developed a Spanish-language version of the 27-item DFS for use in Spain [17]. A second group has developed a Spanish version consisting of 26 of the items, also for use in Spain [35]. When we began our project, there were no published Spanish versions of the 20-item version. However, after our data collection was nearly complete, Lago-Médez and colleagues reported on a Spanish version of the measure, developed for use in Spain [16]. Lago-Médez and colleagues [16] correlated DFS sum scores with Corah's original DAS, finding evidence for the construct validity of the DFS. However, they did not perform reliability analyses.

A third English-language questionnaire has been developed to measure dental injection phobia in greater detail (Needle Survey, NS) [36]. The NS consists of 18 items previously found to have good reliability and validity representing cognitions about dental injections, including general fear of injection and pain, fears related to local anesthesia, fear of acquiring a disease as a result of the injection, and fear of sustaining an injury as a result of the injection. The scale has been found to have good construct validity in a university sample [36]. In a second study, adults who met DSM-IV criteria for specific phobia of dental injections scored higher on the NS than did those described in the university sample, providing additional evidence of the scale's construct validity [37]. There have been no previous attempts to translate the NS into Spanish.

In summary, while there are valid measures of dental fear in English, there is a dearth of Spanish-language versions for use with Hispanics. Therefore, we translated the MDAS, DFS and NS into Spanish and assessed their psychometric properties in three samples of Hispanics: Spanish-speaking adults attending Hispanic community festivals, college students in an area whose population is heavily Hispanic, and Hispanic patients receiving care in a dental clinic. Since English-speaking persons also attended the community festivals and college, we also elected to obtain data on the English versions of these measures in these two populations, which permitted us to compare the performance of the Spanish and English versions in the same populations.

## Methods

Ethical review boards at the University of Washington and Heritage University approved the studies.

### Participants

Data were collected from three groups of participants. For an overall examination of the Spanish-language versions of the fear questionnaires, we recruited participants from two community festivals which celebrated Hispanic culture. One festival took place in Seattle, an urban city, and the second festival took place in a largely-Hispanic area in rural Eastern Washington. These participants gave oral consent. In order to gather test-retest data, a second group of participants included Spanish- and English-speaking college students from Heritage University, a university in a largely-Hispanic area of Eastern Washington. While the language of instruction at the University is English, many students speak Spanish. These participants gave oral consent. The third group of participants included Hispanic patients at a dental clinic in rural Eastern Washington in order to examine the criterion validity of the Spanish-language measures. The adult patients gave written consent. For patients younger than 18, parents provided written consent and the minor patients provided written assent. Summary information about the English- and Spanish-speaking participants is shown in Table 1.

**Table 1:** Sample Characteristics

Sample Source	Number of Participants	Age Range	Mean Age, (SD), Median Age	Percent Female
English-speaking Attendees at Community Festivals	136	18–77	33.6 (13.7) 33.0	65%
Spanish-speaking Attendees at Community Festivals	213	18–88	37.5 (13.3) 35.0	71%
English-speaking College Students	58	19–57	28.7 (10.4) 25.0	60%
Spanish-speaking College Students	31	18–41	24.5 (5.9) 22.0	81%
Spanish-speaking Dental Patients	100	12–62	27.6 (10.9) 27.0	65%

### Materials

We compiled two questionnaires, each of which included the MDAS, DFS, NS, and demographic questions in either English or Spanish. The English language questionnaires were translated into Spanish by a professional translator and back-translated. Since we were focusing our data collection in rural Eastern Washington, an area with a large number of Hispanic residents, a bilingual individual from that area made minor changes to make the Spanish more consistent with local usage. During the first administration of the Spanish questionnaire, some participants found the translated version of one DFS item ("Transpiro", for "I perspire") to be too abstract. After consultation with bilingual experts, we replaced the original Spanish word for "I perspire" with a simpler word ("Sudo"). Subsequent participants did not express any difficulty with the simpler word. The Spanish versions of the MDAS and NS are included in the appendix. For copyright reasons, the Spanish version of the DFS is not included here. Interested persons may contact the author for more information.

### Procedures

At the community festivals, we recruited both Spanish and English speaking participants to provide comparisons between languages. Since our primary interest was in gathering Spanish data, if a potential participant was bilingual, he/she was asked to complete the Spanish language questionnaire. A small number of potential Spanish participants, primarily older, were turned away because of literacy concerns. A total of 136 participants completed the English questionnaire, while 213 completed the Spanish questionnaire. Participants received a gift card for a supermarket as an incentive.

In the college student sample, we first recruited English-speaking students to complete the English questionnaire twice in English, about 1 to 3 weeks apart. Fifty eight students completed the questionnaire in English. Thirty eight of them completed the questionnaire both times, 17 only completed the first questionnaire, and 3 only completed the second questionnaire. In a different academic year, we recruited Spanish-speaking students to complete the Spanish version twice, about 1 to 3 weeks apart. Thirty one students completed the Spanish version of the questionnaire. One student only completed the first questionnaire, and the remaining 30 completed the questionnaire at both administrations. Students were offered the chance of winning a gift certificate at the University bookstore as an incentive.

In the third sample, patients who had dental appointments in a community clinic on the days when the research assistant was present, and who were able to read the Spanish questionnaire, were invited to participate. Recruitment continued until 100 patients agreed to participate. Adolescent and adult patients completed the Spanish version of the questionnaire and indicated what kind of dental treatment they had received. Due to low numbers for some of the specific dental treatments, the patients were categorized according to whether they had received invasive treatment requiring dental injection (restorations, extractions, root canals) or not (examination, X-rays, cleaning). Thirty-six percent stated that they had received one of the invasive treatments, 41% stated that they had only received one or more of the non-invasive treatments, and the remaining 23% did not indicate what their dental treatment had been. The research assistant rated the patients' observed anxiety using Corah's Interval Scale of Anxiety Response (ISAR) [38]. Patients received a gift card for a supermarket as an incentive. Due to clinic procedures, patients completed the questionnaire after the dental treatment.

### Analyses

Questionnaire data were entered into an Excel data base and checked for accuracy. If a participant gave two answers to an item, the mean value was substituted. No other changes were made to the original data. Only completed questionnaires were included in each analysis. Analyses were done in SPSS Version 14.0 (SPSS, Inc., Chicago, IL). In the student samples, scores from the first questionnaire completed were used for summary statistics. Cronbach's alpha was used to determine internal reliability. Intra-class correlations (two-way mixed) were used to compute test-retest reliability. Construct validity was examined by inter-correlating the three measures, since they were presumed to be tapping similar underlying constructs related to dental fear [39]. Criterion validity was assessed by correlating patient scores on the measures and the observer's ratings. Spearman's rho was used for correlations of fear measures, since the distributions of these measures were skewed (most participants had low fear levels). In the dental patient sample, the correlation analyses between observer ratings and patient questionnaire scores included all patients who had completed the questionnaires for the two scales measuring general dental fear (MDAS and DFS). Since the third scale (NS) is specific to dental injections, only patients who had experienced a dental injection on the day of data collection were included in the analysis correlating this measure with the observer ratings.

## Results

Table 2 presents the summary statistics for the three fear measures in each of the samples. Table 3 presents the reliability analyses for the three measures. As seen in this table, the alphas of the Spanish measures were high in each of the three Spanish-language samples (ranging from 0.80 to 0.96) and were similar to those found in the two English samples. This provides evidence for the internal reliabilities of the Spanish versions. Table 3 also includes the intraclass correlations for the Spanish and English scales, based on the college student samples. These were high for the Spanish DFS and NS (0.86 and 0.94, respectively), providing evidence for the high test-retest reliability of these two Spanish measures. However, the value for the MDAS was lower (0.69). Because the test-retest reliability for the Spanish MDAS was lower than that found for the English version (0.69 vs. 0.94), we performed two additional analyses on the Spanish version of this scale. First, an item analysis found that deleting any of the first four items would result in lowering the alpha to 0.78–0.84, compared with the original 0.85 (deleting the fifth item would result in an increase in alpha from 0.85 to 0.86). We next compared each item across the two administrations, using intra-class correlations. This analysis revealed that the third item ("If you were about to have a tooth drilled, how would you feel?") had an intra-class correlation of 0.51 (95% CI = -0.03 – 0.77); there were no other items for which the confidence interval included zero.

**Table 2:** Descriptive Statistics for Modified Dental Anxiety Scale (MDAS), Dental Fear Survey (DFS), and Needle Survey (NS) by Sample

Sample	MDAS Mean (SD)	MDAS Median	MDAS Range	DFS Mean (SD)	DFS Median	DFS Range	NS Mean (SD)	NS Median	NS Range
English-speaking Community	12.64 (4.94)	12.00	5–25	44.11 (16.61)	43.00	20–89	38.09 (14.54)	36.50	18–84
Spanish-speaking Community	13.05 (4.94)	12.00	5–25	39.30 (15.37)	37.00	20–93	38.30 (15.30)	35.00	18–90
English-speaking College Students	13.47 (5.94)	12.00	5–25	44.32 (20.51)	38.00	20–89	36.42 (15.98)	35.00	18–90
Spanish-speaking College Students	11.87 (5.03)	13.00	5–19	40.27 (13.31)	39.50	21–63	36.55 (13.60)	31.00	20–65
Spanish-speaking Patients	11.06 (4.49)	10.00	5–21	33.39 (14.69)	27.00	20–80	37.90 (16.85)	34.00	18–85

**Table 3:** Reliability Analyses for Modified Dental Anxiety Scale (MDAS), Dental Fear Survey (DFS), and Needle Survey (NS) by Sample

Sample	MDAS Alpha	MDAS Intraclass Correlation (95% CI)	DFS Alpha	DFS Intraclass Correlation (95% CI)	NS Alpha	NS Intraclass Correlation (95% CI)
English-speaking Community	0.86		0.96		0.92	
Spanish-speaking Community	0.83		0.95		0.92	
English-speaking College Students	0.91	0.94 (0.87–0.97)	0.97	0.94 (0.88–0.97)	0.94	0.88 (0.73–0.95)
Spanish-speaking College Students	0.85	0.69 (0.34–0.85)	0.92	0.86 (0.68–0.97)	0.92	0.94 (0.86–0.97)
Spanish-speaking Patients	0.80		0.96		0.94	

Tables 4, 5 and 6 present the construct validity data in the community, student, and patient samples, respectively. The construct validity for the measures was assessed by correlating the three measures with each other in each sample. In each case, the three measures were significantly correlated with one another, with p values < 0.01 in two cases and < 0.001 in every other case.

**Table 4:** Correlations Between Modified Dental Anxiety Scale (MDAS), Dental Fear Survey (DFS), and Needle Survey (NS) in the English-speaking and Spanish-speaking Community Samples

	English DFS	English NS	Spanish DFS	Spanish NS
English MDAS	0.65*** n = 115	0.44*** n = 121		
English DFS		0.63*** n = 112		
Spanish MDAS			0.67*** n = 130	0.47*** n = 151
Spanish DFS				0.56*** n = 117

* = p < 0.05

** = p < 0.01

*** = p < 0.001

**Table 5:** Correlations Between Modified Dental Anxiety Scale (MDAS), Dental Fear Survey (DFS), and Needle Survey (NS) in the English-speaking and Spanish-speaking College Student Samples

	English DFS	English NS	Spanish DFS	Spanish NS
English MDAS	0.83*** n = 40	0.53*** n = 40		
English DFS		0.68*** N = 36		
Spanish MDAS			0.65*** n = 26	0.50*** n = 29
Spanish DFS				0.69*** n = 25

* = p < 0.05

** = p < 0.01

*** = p < 0.001

**Table 6:** Correlations Between Modified Dental Anxiety Scale (MDAS), Dental Fear Survey (DFS), and Needle Survey (NS) in the Spanish-speaking Dental Sample

	MDAS	DFS	NS
MDAS		0.66*** n = 73	0.36** n = 82
DFS			0.44*** n = 64

* = p < 0.05

** = p < 0.01

*** = p < 0.001

Criterion validity was assessed in the sample of dental patients. Behavioral ratings of anxiety (ISAR) were made for 97 of the 100 patients. In these ratings, higher scores indicated greater observable anxiety. The correlations between the ISAR and the two general dental fear measures were r = 0.37 for the MDAS (p < 0.001) and r = 0.39 for the DFS (p = 0.001). The correlation between the ISAR and the NS was r = 0.45 (p = 0.012) for patients who had received a dental injection as part of their dental treatment.

## Discussion

This study is the first to examine the psychometrics of Spanish versions of the MDAS and the NS. We also developed and assessed a new translation of the 20-item DFS for Hispanics.

We found very good evidence for the internal reliabilities of all three Spanish versions of the fear measures, with coefficients ranging from 0.80 to 0.96. We also found good evidence for the test-retest reliability of the DFS and NS. The one anomalous finding was that the test-retest reliability for the Spanish version of the MDAS was lower. This was surprising, since the summary statistics for all three measures were similar for the English and Spanish students, and the test-retest for the English version of the MDAS was acceptable. Although the item analysis of the Spanish MDAS indicated that all five items were measuring the same construct, the comparison of each item across both administrations indicated that one item ("If you were about to have a tooth drilled, how would you feel?") was not answered consistently, contributing to the lower test-retest value. As a result of these analyses, we subsequently had three additional bilingual translators look at this item, and there is disagreement across all six of our translators over whether the Spanish translation for "drilled" is accurate.

The significant correlations found between the three fear measures in the Spanish samples provide evidence of their construct validity. In addition, the similarities in magnitude seen in the Spanish correlations when compared with the English results in both the community and student samples provide evidence that the Spanish versions appear to be behaving similarly to their English counterparts. Given that the English versions have established validity, these similarities across language versions provide additional evidence for the construct validity of the Spanish language versions.

In each sample, the correlations between the MDAS and DFS were higher than those between the NS and either the MDAS or the DFS. While many fearful patients express anxiety about dental injections, it is important to keep in mind that dental fear presents heterogeneously, and not all fearful patients are anxious about injections [8]. It is likely that our samples included individuals who had fears of some dental stimuli but not necessarily of dental injections, resulting in lowered correlations between the two measures of general dental fear and the NS, which specifically targets concerns about dental injections.

In terms of criterion validity, the relationships between the participants' self-report and the observer rating were significant for both of the general fears measures, as well as for the NS for those patients who experienced a dental injection. According to Cohen [40], the magnitudes of the correlations are moderate. Our results are similar to those reported by Corah [12], who found correlations of 0.41 and 0.42 between the original DAS and dentists' ratings of anxiety, and Neverlien and Johnsen [41], who found the DAS and observer ratings of behavioral relaxation to have a correlation of 0.44. In the future, it would be desirable to explore the criterion validity of these measures by the contrasted group method, in which the scores of Hispanic patients previously-identified as fearful are compared with the scores of Hispanic patients who are not identified as fearful.

There are several reservations about our study. For practical purposes, we used samples of convenience in this study, and it would be desirable to replicate the results using other samples of Hispanics. As described above, the test-retest value found for the Spanish MDAS was lower than expected, perhaps due to the translation of one item. In addition, this study used a written questionnaire to measure dental fear. We found that some Hispanics could not read and therefore could not participate in the study. It is possible that the Hispanics whom we turned away might have different attitudes towards dental fear. Other studies of Hispanics have developed alternatives to written questionnaires when literacy rates are lower, such as those described by Boiko and colleagues [42]. It would be desirable to be able to include such alternatives in the future, to be able to gather data from more representative samples of Hispanics.

## Conclusion

In sum, we found evidence for the internal reliability, construct validity, and criterion validity for the Spanish versions of the MDAS, DFS, and NS. We also found evidence for the test-retest reliability of the DFS and NS. On the other hand, the test-retest reliability of the Spanish MDAS was lower than expected.

## Competing interests

The authors declare that they have no competing interests.

## Authors' contributions

TC and SEC were the primary contributors to the design of the study. TC, MAC, LJG and SEC collected data. All authors contributed to the data analyses. TC drafted the manuscript. All authors read and approved the final manuscript.

## Appendix

Spanish MDAS Items:

1. ¿'Cómo se sentiría si tuviera que ir a su dentista para un tratamiento mañana?

2. ¿'Cómo se sentiría si estuviera sentado/a en la sala de espera (esperando por el tratamiento)?

3. ¿'Cómo se sentiría si estuvieran a punto de agujerarle un diente?

4. ¿'Cómo se sentiría si estuvieran a punto de quitarle el sarro de los dientes y pulírselos?

5. ¿'Cómo se sentiría si estuvieran a punto de ponerle una inyección de anestesia local en su encía, sobre uno de los dientes de arriba de la parte de atrás de su boca?

Spanish NS Items:

Con respecto a las inyecciones dentales, yo creo que:

1. La aguja anestésica puede golpear un nervio o algo y herirme.

2. Nada es tan doloroso como una aguja en mi boca.

3. Soy muy difícil de anestesiar.

4. Si mi garganta se adormece por la inyección, no podré respirar.

5. Si mi garganta se adormece por la inyección, no podré ingerir.

6. Ver la aguja es aterrorizante.

7. Soy sensible a la Novocaína y puede hacerme daño.

8. El adormecimiento no desaparecerá.

9. Sentir que la aguja pica la piel es la peor parte.

10. El dentista puede resbalarse y lastimarme.

11. Solo la idea de una aguja penetrando mi cuerpo me causa terror.

12. No se por que las agujas me aterrorizan.

13. Inyectar químicos desconocidos en mi cuerpo me causa miedo.

14. Me da miedo ver la aguja acercarse a mi boca.

15. La aguja no esta limpia y puedo contraer una enfermedad.

16. La aguja no esta limpia y puedo contraer una infección.

17. Es posible que me mueva y haga que el dentista me lastime.

18. Pueda que sangre como resultado de la inyección.

## Pre-publication history

The pre-publication history for this paper can be accessed here:


